# White matter tracts involved in subcortical unilateral spatial neglect in subacute stroke

**DOI:** 10.3389/fneur.2022.992107

**Published:** 2022-09-30

**Authors:** Seungwoo Cha, ByeongChang Jeong, Myungwon Choi, Sohyun Kwon, Stephanie Hyeyoung Lee, Nam-Jong Paik, Won-Seok Kim, Cheol E. Han

**Affiliations:** ^1^Department of Rehabilitation Medicine, Seoul National University College of Medicine, Seoul National University Bundang Hospital, Seongnam, South Korea; ^2^Department of Electronics and Information Engineering, Korea University, Sejong, South Korea; ^3^Interdisciplinary Graduate Program for Artificial Intelligence Smart Convergence Technology, Korea University, Sejong, South Korea

**Keywords:** stroke, subcortical, unilateral spatial neglect, atlas-based lesion overlapping analysis, white matter tract

## Abstract

**Background:**

Unilateral spatial neglect (USN) is common and associated with poor motor and cognitive outcomes as well as impaired quality of life following stroke. Traditionally, the neural substrates underlying USN have been thought to be cortical areas, such as the posterior parietal cortex. However, patients with stroke involving only subcortical structures may also present with USN. While only a few studies have reported on USN in subcortical stroke, the involvement of white matter tracts related to brain networks of visuospatial attention is one possible explanation for subcortical neglect. Therefore, this study aimed to investigate which specific white matter tracts are neural substrates for USN in patients with subcortical stroke.

**Methods:**

Twenty-two patients with subcortical stroke without cortical involvement who were admitted to the Department of Rehabilitation Medicine at Seoul National University Bundang Hospital were retrospectively enrolled. Nine subjects were subclassified into a “USN(+)” group, as they had at least two positive results on three tests (the Schenkenberg line bisection test, Albert's test, and house drawing test) and a score of 1 or higher on the Catherine Bergego scale. The remaining 13 subjects without abnormalities on those tests were subclassified into the “USN(–)” group. Stroke lesions on MRI were manually drawn using MRIcron software. Lesion overlapping and atlas-based analyses of MRI images were conducted. The correlation was analyzed between the overlapped lesion volumes with white matter tracts and the severity of USN (in the Albert test and the Catherine Bergego scale).

**Results:**

Lesions were more widespread in the USN(+) group than in the USN(–) group, although their locations in the right hemisphere were similar. The atlas-based analyses identified that the right cingulum in the cingulate cortex, the temporal projection of the superior longitudinal fasciculus, and the forceps minor significantly overlapped with the lesions in the USN(+) group than in the USN(–) group. The score of the Catherine Bergego scale correlated with the volume of the involved white matter tracts.

**Conclusion:**

In this study, white matter tracts associated with USN were identified in patients with subcortical stroke without any cortical involvement. Our study results, along with previous findings on subcortical USN, support that USN may result from damage to white matter pathways.

## Introduction

The prevalence of unilateral spatial neglect (USN) following stroke is approximately 30% (1). Although patients are thought to recover from USN over time, it can persist for more than 1 year after onset (2, 3). The presence of USN is associated with poor motor and cognitive outcomes as well as impaired quality of life in patients with stroke (4, 5). Furthermore, the severity of USN is a predictor of functional outcomes after a right hemispheric stroke (6). In addition, USN increases the risk of falls and the burden on caregivers (7, 8).

Traditionally, functional brain areas related to USN have been thought to be cortical areas, such as the posterior parietal cortex, based on an early observational study (9). However, anatomo-clinical correlation studies based on structural brain imaging have shown that the inferior parietal lobule is closely associated with USN symptoms (10). In addition, other studies have reported that damage to the right superior temporal gyrus or the ventrolateral prefrontal cortex is correlated with USN (11–13).

However, USN has also been reported in patients with subcortical stroke without the involvement of cortical structures (3). In addition to the possible role of impairments in subcortical gray matter in USN (14), a few previous studies have reported that white matter tracts involved in visuospatial networks can also induce USN. A study in 140 patients with acute cortical and subcortical strokes reported that the superior longitudinal fasciculus, the inferior occipitofrontal fasciculus, and the superior occipitofrontal fasciculus were associated with USN (15). Another study of 45 chronic cortical and subcortical stroke cases showed decreased fractional anisotropy in the right superior longitudinal fasciculus (SLF) and the splenium of the corpus callosum on diffusion tensor imaging (16). A recent study of 174 patients with acute cortical and subcortical strokes using connectome-based lesion-symptom mapping analysis revealed that tracts from the right parietal cortex and left or right mesial temporal cortex were strongly associated with USN (17). In addition, Umarova et al. reported that patients with USN after a stroke of the right middle cerebral artery territory exhibited a symptom-correlating reduction of fractional anisotropy in regions connected to the left dorsal attention system (18). From this perspective, Bartolomeo et al. suggested that the white matter pathways connecting frontal and parietal regions may have a crucial role in the pathogenesis of USN (19). However, the aforementioned studies included patients with cortical stroke as well as those with subcortical stroke (15–18) although the involvement of white matter tracts may differ based on whether the lesion is cortical or subcortical. Subcortical stroke may induce pathologic connectivity within or across hemispheres (20); therefore, evaluating neural substrates related to USN focusing on subcortical stroke is worthwhile. In addition, considering that recovery through reorganization of the structure-function relationship may confound the lesion-symptom analysis (17), assessing patients with stroke in the early stages would yield meaningful insights.

Therefore, this study aimed to investigate specific white matter tracts as neural substrates for USN in patients with subcortical stroke without any cortical involvement, using atlas-based white matter involvement along with voxel-based lesion overlapping analyses.

## Materials and methods

### Subjects

We retrospectively reviewed the data of patients admitted to the Department of Rehabilitation Medicine at Seoul National University Bundang Hospital from January 2012 to June 2017. We selectively chose patients with first-ever right hemisphere stroke only involving subcortical brain structures, including the basal ganglia, thalamus, internal capsule, and corona radiata. Among these, we included patients whose records included an initial brain magnetic resonance imaging (MRI) assessment and an evaluation for unilateral spatial neglect (USN) consisting of the Schenkenberg line bisection test (21), Albert's test (22), and the house drawing test. Patients without any abnormalities in the three tests were subcategorized into the “USN(–) group” (*n* = 13). In contrast, those with abnormalities in at least two of three tests (1) omission of two or more whole lines on the left side in the line bisection test; (2) over 70% of lines uncrossed on the left side of the midline in the Albert test (22); and (3) any significant omissions on the left side (such as door, window, roof, chimney, and smoke) in the house drawing test) were further evaluated with the Catherine Bergego scale (23) to find clinically meaningful USN. Then, those with a Catherine Bergego scale score of 1 or higher (24) were subcategorized into the “USN(+) group” (*n* = 9). Trained occupational therapists conducted and recorded the evaluation. Results of the USN evaluation are presented in Supplementary Table 1. This study was approved by the Seoul National University Bundang Hospital Institutional Review Board, which waived the need for informed consent (IRB No. B-1706/401-102).

### Acquisition and preprocessing of MRI data

T1-weighted magnetic resonance (MR) images were acquired using a 3.0 T MRI (Siemens Trio Trim scanner) at Seoul National University Bundang Hospital, using a magnetization-prepared rapid gradient echo sequence (TE/TR/T1=2.32 ms/2.3 s/900 ms; 256 × 256 × 192 matrix for 1 mm^3^ isovoxels). Fluid-attenuated inversion recovery (FLAIR) images were also recorded to identify lesions. Diffusion-weighted imaging (DWI) findings were used in four cases [all in the USN(–) group] where FLAIR images were not available.

To perform our retrospective analyses, we localized the lesions of all subjects into the same standard space. First, the FLAIR image of each subject was linearly co-registered with their T1-weighted image. Second, the T1-weighted image (linearly and non-linearly registered) was normalized to the standardized International Consortium for Brain Mapping (ICBM) template for East Asian brains presented by Statistical Parametric Mapping (SPM). Subsequently, using the parameters obtained from the previous steps, the co-registered FLAIR images were registered and resliced on the ICBM template. More accurate normalization of the images could be obtained using this two-step procedure. Finally, the neuroanatomy specialists (SK and SHL) manually drew lesions onto the aligned images using MRIcron software (25). These region-of-interest (ROI) images in the standardized template were used in analyses. This methodology has been described in detail in a previous study (26). All preprocessing was performed using SPM12 software (27–29).

### Analysis of white matter tract involvement

We analyzed how each patient's lesions affected their white matter tracts. We used the volumetric white matter tracts defined in the white matter atlas generated by Johns Hopkins University (30, 31). It contains 20 tracts in total; however, we included only 11: the anterior thalamic radiation, cingulum in the cingulate cortex, cingulum in the hippocampal area, corticospinal tract, forceps major, forceps minor, inferior fronto-occipital fasciculus, inferior longitudinal fasciculus, superior longitudinal fasciculus, temporal projection of the SLF, and uncinate fasciculus connected to the right hemisphere where the lesions in our patients were located. We then measured the overlapped volume of each individual's lesions. We compared the overlapped volumes between groups through permutation testing (32, 33) since the number of subjects was small, and the distribution of overlapped volumes was not normally distributed (Supplementary Figure 3); specifically, permutation-based ANCOVA (34) was used and adjusted for age and sex, with a permutation number of 1,000. We used a false discovery rate (FDR) procedure for the 11 white matter tracts as a multiple comparison correction (35). In addition, we performed the correlation study between the overlapped volume of white matter tracts and USN severity scores in the USN(+) group: the percentage of total uncrossed lines in the Albert test and the Catherine Bergego scale score. The severity scores were skewed, and the sample size was small; therefore, we used the Spearman correlation coefficients. For the statistical analyses, our in-house codes and the LinStat library (2006b) (36) in a MATLAB (2019a, MathWorks) were used.

### Lesion overlapping analysis

**Figure 1:**
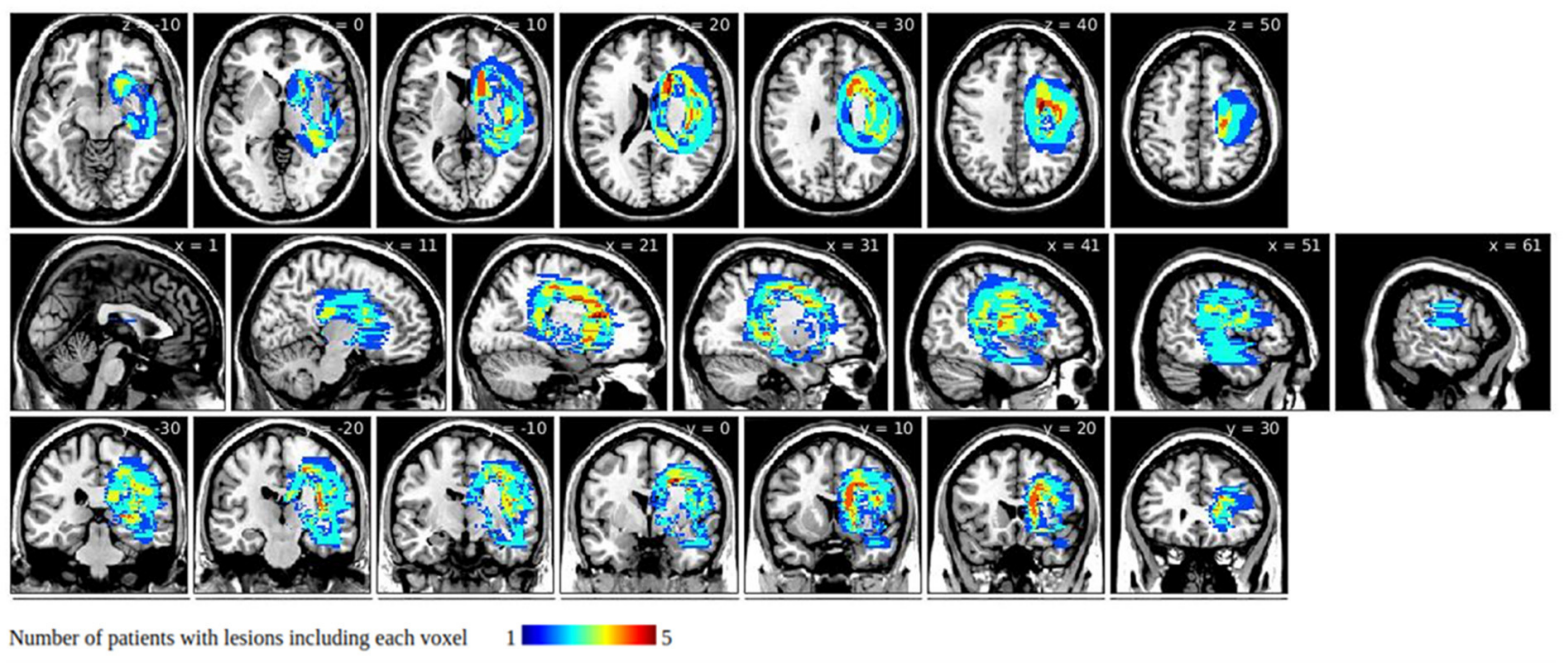
Symptom-related lesions obtained by subtracting group-level unilateral spatial neglect (USN)(–) lesions from group-level USN(+) lesions. Warmer colors indicate larger numbers of subjects with overlapping lesions in the USN(+) group.

We also visualized how group-level lesions overlapped with the 11 white matter tracts. This is a complementary visualization to the statistical analysis mentioned above. This presents an overall involvement of white matter tracts but is not identical to the statistical analysis results. We first extracted the group-level lesions of all patients in the USN(+) and USN(–) groups. We then subtracted the latter from the former to isolate symptom-related lesions. These symptom-related lesions were then overlapped with the 11 white matter tracts in the FMRIB software library v5.0.9 (37) and visualized using BrainNet Viewer (38) and voxel lesion symptom mapping (VLSM, version 2.55; https://aphasialab.org/vlsm/). Regarding the latter, note that we did not perform VLSM but used it to organize our visualization in Figure 1 and Supplementary Figures 1, 2.

## Results

### Subject characteristics

The comparison of characteristics between the USN(+) group and the USN(–) group is presented in Table 1. The mean age of patients in the USN(+) group was significantly lower than that of the patients in the USN(–) group (54.4 vs. 69.2 years). In addition, the median lesion volume was significantly higher in USN(+) group (68,328 vs. 21,416 mm^3^). There were no significant differences in sex, stroke type and location, days from onset to imaging, or days from onset to evaluation between the two groups.

**Table 1:** Comparing the baseline characteristics.

	**Unilateral spatial neglect**	**No unilateral spatial neglect**	***Test statistics***	***p*-value**
	**(*n* = 9)**	**(*n* = 13)**		
**Age, years**	54.4 ± 16.3	69.2 ± 12.2	−2.437^a^	0.024*
**Male, n (%)**	6 (66.7%)	10 (76.9%)	0.282^b^	0.595
**Stroke type, n (%)**			3.010^b^	0.083
Ischemic	1 (11.1%)	6 (46.2%)		
Hemorrhagic	8 (88.9%)	7 (57.1%)		
**Stroke location, n (%)**			2.996^b^	0.558
Basal ganglia	6 (66.7%)	5 (38.5%)		
Thalamus	2 (22.2%)	3 (23.1%)		
Internal capsule	1 (11.1%)	2 (15.4%)		
Basal ganglia & internal capsule	0 (0%)	2 (15.4%)		
Corona radiata	0 (0%)	1 (7.7%)		
**Lesion volume, median (IQR)**	68328 (176060)	21416 (22044)	6.095^c^	0.024*
**Onset to imaging, days, median (IQR)**	0 (1.5)	1 (1)	51.0^d^	0.647
**Onset to evaluation, days, median (IQR)**	14 (10)	14 (7.5)	76.5^d^	0.235

*Statistically significant results. IQR, interquartile range.

^*a*^t-statistics of 2-sample t-test, ^*b*^χ^2^ value of Chi-Square test, ^*c*^F-statistics of ANCOVA controlling for the effects of age and sex, ^*d*^U statistics of Mann-Whitney.U test.

Clinical characteristics for each patient are shown in Supplementary Table 1.

### Lesion overlapping results

Group-level lesions in the USN(+) and the USN(–) group are presented in Supplementary Figures 1, 2, respectively. The overlapped lesions of both groups were similarly located in the right hemisphere; however, the lesions in the USN(+) group were more widespread than those in the USN(–) group.

Group-level lesions of the USN(–) group subtracted from those of the USN(+) group are presented in Figure 1. They involved areas around the caudate nucleus, the internal capsule, and the corpus callosum, but they spared the thalamus, putamen, globus pallidus, and the corona radiata.

### White matter tracts involvement

**Figure 2:**
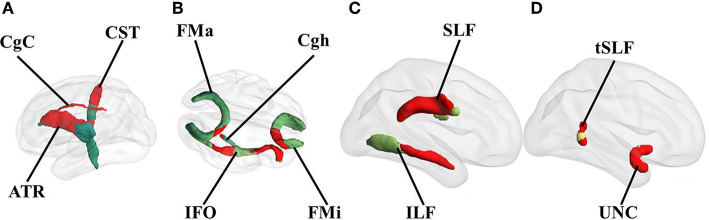
Overlap of white matter tracts with the identified symptom-related lesions. Red represents the overlapping parts of the white matter tracts with the lesions. Only the involved tracts are shown. All overlapping tracts located in the right hemisphere. **(A)** medial view, **(B)** transverse view, and **(C,D)** lateral view of the right hemisphere. CST, corticospinal tract; ATR, anterior thalamic radiation; CgC, cingulum in the cingulate cortex; CgH, cingulum in the hippocampal area; FMa, forceps major; FMi, forceps minor; IFO, inferior fronto-occipital fasciculus; SLF, superior longitudinal fasciculus; ILF, inferior longitudinal fasciculus; UNC, uncinate fasciculus.

The overlap with the white matter tracts revealed the involvement of the right anterior thalamic radiation, corticospinal tract, inferior longitudinal fasciculus, inferior fronto-occipital fasciculus, uncinate fasciculus, superior longitudinal fasciculus and its temporal projection, cingulum in the cingulate cortex, and the slight involvement of the cingulum in the hippocampal area, forceps major, and the forceps minor (Figure 2). However, these diagrams are a rough illustration of the overlapping lesion locations; therefore, they cannot represent the statistical difference between the groups.

When we compared white matter tract involvement using the overlapped volumes between the individual's lesions and volumetric white matter tracts while controlling the effects of age and sex using permutation-based ANCOVA, we observed widespread overlaps on the tracts in the USN(+) group (Table 2). The statistical analysis ruled out white matter tracts that were involved to similar degrees in both groups. We observed that the right cingulum in the cingulate cortex [*F* (1, 20) = 5.074, FDR-adjusted *p* = 0.026; all *p*-values in this and the following section are FDR-adjusted], the temporal projection of the superior longitudinal fasciculus (*F* = 6.724, *p* = 0.026), and the forceps minor (*F* = 3.468, *p* = 0.026) were significantly affected. In other words, the volume of the involved white matter tracts was greater in the USN(+) group than in the USN(–) group after adjusting for age and sex. The anterior thalamic radiation (*p* = 0.077), superior longitudinal fasciculus (*p* = 0.081), and forceps major (*p* = 0.077) were marginally affected. Detailed results regarding the differences between both groups are listed in Supplementary Figure 3 as a histogram.

**Table 2:** Overlap (mm^3^) of the lesions in each group with the major white matter tracts.

**White matter tractography atlas**	**Unilateral spatial neglect (*****n*** = **9) median [25...75%]**		**No unilateral spatial neglect (*****n*** = **13) median [25...75%]**		***F*-statistics**	**uncorrected *p*-value**	**FDR-adjusted *p-*value**
Anterior thalamic radiation R	2720	[182…4868]	480	[0…1722]	4.815	0.035	0.077
Corticospinal tract R	1664	[1352…2904]	1216	[496…1588]	2.795	0.115	0.141
**Cingulum (cingulate gyrus)** R	0	[0…164]	0	[0…0]	5.074	0.006	0.026*
Cingulum (hippocampus) R	0	[0…14]	0	[0…0]	2.858	0.101	0.139
Forceps major	0	[0…184]	0	[0…0]	4.255	0.028	0.077
**Forceps minor**	16	[0…442]	0	[0…0]	3.468	0.007	0.026*
Inferior fronto-occipital fasciculus R	1976	[284…3872]	72	[0…2182]	2.482	0.143	0.157
Inferior longitudinal fasciculus R	16	[0…1638]	0	[0…42]	3.511	0.083	0.130
Superior longitudinal fasciculus R	4000	[1004…7256]	88	[0…658]	4.859	0.044	0.081
Uncinate fasciculus R	176	[0…442]	0	[0…190]	1.792	0.184	0.184
**Superior longitudinal fasciculus (temporal projection) R**	0	[0…26]	0	[0…0]	6.724	0.004	0.026*

p-values were calculated through permutation-based ANCOVA controlling for the effects of age and sex.

*Statistically significant results after the FDR procedure.

### Correlation between the white matter tract involvement and USN severity

We performed a correlation study between the significantly affected white matter tracts in the USN(+) group: the cingulum in the cingulate cortex, and the forceps minor, the temporal projection of the superior longitudinal fasciculus and USN severity (the percentage of total uncrossed lines in the Albert test and the Catherine Bergego scale score). Specifically, we presented the correlation between the overlapped volume of the affected white matter tracts and two severity measures of USN (Supplementary Table 2). The percentage of the total uncrossed lines was not significantly correlated with the white matter tracts involvement. However, the score of the Catherine Bergego scale was significantly correlated with the white matter tracts involvement (*r* = 0.822, *p* = 0.012 for the cingulum in the cingulate cortex; *r* = 0.897, *p* = 0.002 for the forceps minor; *r* = 0.895, *p* = 0.003 for the temporal projection of the superior longitudinal fasciculus). The scatter plots are illustrated in Supplementary Figure 4.

## Discussion

This study investigated white matter tract involvement as a structural neural substrate of USN in subcortical stroke using atlas-based lesion overlapping analyses. The study population included only patients with subcortical stroke and without cortical lesions. Thus, the results of this study exclude the effects of cortical involvement in USN. The USN(+) group had more wide spread lesions showing greater lesion volumes than the USN(–) group. The specific white matter tracts affected by USN were the right cingulum in the cingulate cortex, the temporal projection of the superior longitudinal fasciculus, and the forceps minor. Regarding USN severity, the score of the Catherine Bergego scale correlated with the involvement of white matter tracts, while a total of uncrossed lines in the Albert test did not. This supports that USN may result from the disconnection of white matter tracts (19).

The cingulum, as a part of the limbic system, consists of longer fibers (connecting the medial temporal regions and sub-genual frontal area) and shorter fibers (connecting the adjacent medial parietal and frontal lobes) (39). The dorsal cingulum isassociated with attention and executive functions, whereas the temporal cingulum is associated with learning and episodic memory (40). A study on healthy subjects revealed that the cingulum could contribute to the coordination of spatial attention (41). Although the cingulate cortex has been reported to be associated with symptoms of USN following stroke (42), few studies have investigated the association between the cingulum (as white matter tracts) and USN. A study assessing patients with motor neglect reported that damage to the cingulum is associated with motor neglect, possibly by inducing unilateral dysfunction of the medial motor system (43). Our current results are consistent with previous findings that the cingulum in the cingulate cortex is closely associated with USN in subacute subcortical stroke. Since only visuospatial neglect was evaluated in our study, further studies investigating various types of USN are warranted.

The superior longitudinal fasciculus links the frontal cortex with the temporal, parietal, and occipital lobes and plays a major role in language, attention, memory, and emotions (44). Previous studies have demonstrated that damage to the superior longitudinal fasciculus is consistently found in patients with USN following stroke (15, 16, 45). Moreover, different loci of the involved superior longitudinal fasciculus were suggested to have a role in diverse USN phenotypes (46). Similarly, our current results reveal that the superior longitudinal fasciculus is closely associated with USN; however, the temporal projection of the superior longitudinal fasciculus was more specifically affected, and future studies on the correlation between lesions or locations of white matter tracts using more sensitive imaging modalities such as diffusion tensor imaging could support this.

The corpus callosum is the largest neural pathway that connects the two cerebral hemispheres (47). The forceps major receives fibers from the splenium of the corpus callosum connecting the occipital lobes, whereas the forceps minor receives fibers from the genu of the corpus callosum connecting the frontal lobe regions (48). Previous studies on USN observed reduced fractional anisotropy in the splenium of the corpus callosum as well as in the forceps major, enabling interhemispheric communication (16, 49). A recent study revealed that the induction of the right posterior parietal cortex dysfunction provoked USN symptoms and increased resting state functional connectivity between the right posterior parietal cortex and the left superior temporal gyrus (50). In addition, this effect correlated with fractional anisotropy in the posterior corpus callosum, implying that callosal anisotropy could predict changes in the attentional network. In line with these studies, our current results reveal a significant association between the forceps minor and USN.

Increasing age in stroke patients increases risk and USN severity (51, 52). Brain atrophy, white matter disease, or prior ischemic history, common in older patients, were suggested as possible explanations. However, sex was not associated with frequency, severity, or types of USN (52, 53). In addition, there was no significant difference in visuospatial perception between patients with hemorrhage and demographically matched patients with infarction (54). In our study, the lesion volume of involved white matter tracts was compared with adjustment for age and sex, revealing greater involvement in the USN(+) group than in the USN(–) group. However, considering that only one patient had an infarction in the USN(+) group, future studies involving more ischemic stroke patients with USN would further analyze the effect of stroke type on USN.

Previous studies have reported that USN in subcortical stroke may be closely associated with cortical hypoperfusion. Hillis et al. reported that in 14 subjects with only subcortical lesions, concurrent cortical hypoperfusion was strongly associated with USN rather than the lesion site; a lesion comparison analysis between cases and controls was, however, not conducted (55). Similarly, another study in 50 patients with acute right subcortical infarcts found, using perfusion imaging, that hypoperfusion of the right superior temporal gyrus or right angular gyrus rather than the subcortical infarct itself was strongly associated with USN (56). However, this study used Brodmann area landmarks instead of a voxel-based analysis, and specific atlas-based white matter tracts could not be identified. Therefore, future studies using voxel-based lesion analyses with additional perfusion imaging would be valuable to determine the exact relationship between lesions involving subcortical white matter tracts and cortical hypoperfusion in the genesis of USN.

Our study has several limitations. First, the mean age of the patients in the USN(+) group was significantly lower than that of the USN(–) patients, possibly due to the insufficient number of included subjects. However, the results were controlled for age and sex using a permutation-based ANCOVA. Second, because the number of subjects was too small, traditional voxel-based lesion symptom mapping (VLSM) could not be applied due to low statistical power. Instead, we used a simple lesion overlapping analysis. Third, our method of white matter tract involvement analysis might have been somewhat oversimplified. Because we did not have diffusion tensor images, a specific analysis of white matter deterioration was not performed. Instead, the JHU white matter atlas was used. However, actual white matter tracts may be simply deformed because of the lesion which was not completely disconnected. In such cases, the analysis may yield inaccurate results. In addition, we measured the overlapping lesion and white matter tract volumes. Even when the white matter structures of subjects are captured by the JHU atlas, a higher volume overlap alone may not indicate that the tracts are disconnected. Even with a very small overlap, the tracts may be completely disconnected; on the other hand, even when the overlap volume is large, the small cross-section of tracts might have been preserved and the tracts could thus have been spared. Our analysis did not consider such possibilities; therefore, our findings need to be interpreted with some caution. Finally, our evaluation for USN was limited to egocentric and peripersonal USN types. Therefore, other USN types, such as allocentric, intrapersonal, and motor USN were not investigated. In addition, the score of Catherine Bergego scale was rated by occupational therapists and not the patients; therefore, the anosognosia could not be evaluated. Future studies about white matter tract involvement using more sophisticated and diverse evaluation tools would be valuable.

In conclusion, atlas-based lesion overlapping analyses reveal that white matter tracts, including the right cingulum in the cingulate cortex, the temporal projection of the superior longitudinal fasciculus, and the forceps minor, are closely associated with USN in patients with subacute subcortical stroke without cortical involvement. We also confirmed the previous hypothesis that USN may result from damage to white matter pathways rather than damage to a single cortical region (19) by excluding the possible effects of cortical involvement. With our unique study population, the results of the current study could extend those of previous studies on subcortical USN and present clear evidence of white matter involvement.

## Data availability statement

The original contributions presented in the study are included in the article/Supplementary material, further inquiries can be directed to the corresponding author.

## Ethics statement

This study was approved by the Seoul National University Bundang Hospital Institutional Review Board, which waived the need for informed consent (IRB No. B-1706/401-102). Written informed consent for participation was not required for this study in accordance with the national legislation and the institutional requirements.

## Author contributions

SC, W-SK, CH, and N-JP: conceived and coordinated the study. SK and SL: gathered and classified the clinical and imaging data under the supervision of SC, W-SK, and N-JP. BJ and MC: processed the MRI data and conducted atlas- and voxel-based lesion overlapping analyses under the supervision of CH. BJ, MC, and CH: visualized the results. SC, W-SK, BJ, MC, and CH: drafted the manuscript. All authors participated in the interpretation of the results and reviewing and editing the manuscript.

## Funding

This work was supported by the Ministry of Education of the Government of the Republic of Korea (NRF-2021R1F1A1063342 to CH) and a National Research Foundation of Korea (NRF) grant funded by the Korean government (MSIT) (NRF-2022R1A2C1006046 to W-SK).

## Conflict of interest

The authors declare that the research was conducted in the absence of any commercial or financial relationships that could be construed as a potential conflict of interest.

## Publisher's note

All claims expressed in this article are solely those of the authors and do not necessarily represent those of their affiliated organizations, or those of the publisher, the editors and the reviewers. Any product that may be evaluated in this article, or claim that may be made by its manufacturer, is not guaranteed or endorsed by the publisher.
