# Supplementary material for: White matter tracts involved in subcortical unilateral spatial neglect in subacute stroke
Source: Front Neurol. 2022 Sep 30;13:992107. doi: 10.3389/fneur.2022.992107 (PMC9561922; doi:10.3389/fneur.2022.992107)
Supplement: Supplementary file 1 [file Data_Sheet_1.docx]

**Supplementary Materials**

**Table S1.** Clinical characteristics of patients

|  | USN | Age | Sex | Type | Schenkenberg  line bisection test,  lines omitted  (left/middle/right) | Albert test | | Drawing test | Catherine Bergego scale | Lesion volume,  mm^3^ |
| --- | --- | --- | --- | --- | --- | --- | --- | --- | --- | --- |
|  |  |  |  |  |  | Total uncrossed lines, % | Uncrossed lines on the left, % |  |  |  |
| P1 | (+) | 86 | F | Infarction | 2 / 0 / 0 | 72.2% | 85.7% | Neglect pattern | 6 | 3,792 |
| P2 | (+) | 30 | M | Hemorrhage | 5 / 2 / 1 | 61.1% | 100% | Neglect pattern | 9 | 37,240 |
| P3 | (+) | 58 | F | Hemorrhage | 2 / 0 / 0 | 0 | 0 | Neglect pattern | 5 | 37,784 |
| P4 | (+) | 45 | M | Hemorrhage | 3 / 0 / 0 | 58.3% | 92.3% | Neglect pattern | 12 | 69,192 |
| P5 | (+) | 49 | M | Hemorrhage | 6 / 6 / 0 | 86.1% | 63.2% | Neglect pattern | 13 | 33,288 |
| P6 | (+) | 67 | F | Hemorrhage | 6 / 6 / 0 | 8.3% | 100% | Neglect pattern | 21 | 257,720 |
| P7 | (+) | 47 | M | Hemorrhage | n.a. | 80.6% | 70.6% | Neglect pattern | 17 | 68,328 |
| P8 | (+) | 64 | M | Hemorrhage | 6 / 4 / 0 | 52.8% | 92.3% | Neglect pattern | 25 | 322,328 |
| P9 | (+) | 44 | M | Hemorrhage | 6 / 6 / 0 | 11.1% | 100% | Neglect pattern | 23 | 197,176 |
| C1 | (-) | 62 | M | Hemorrhage | 0 / 0 / 0 | 0 | 0 | No neglect | 0 | 77,064 |
| C2 | (-) | 89 | M | Infarction | 0 / 0 / 0 | 0 | 0 | No neglect | 0 | 11,104 |
| C3 | (-) | 72 | M | Infarction | 0 / 0 / 0 | 0 | 0 | No neglect | 0 | 7,760 |
| C4 | (-) | 72 | M | Hemorrhage | 0 / 0 / 0 | 0 | 0 | No neglect | 0 | 21,824 |
| C5 | (-) | 65 | M | Hemorrhage | 0 / 0 / 0 | 0 | 0 | No neglect | 0 | 24,184 |
| C6 | (-) | 60 | F | Hemorrhage | 0 / 0 / 0 | 0 | 0 | No neglect | 0 | 21,416 |
| C7 | (-) | 57 | M | Hemorrhage | 0 / 0 / 0 | 0 | 0 | No neglect | 0 | 65,456 |
| C8 | (-) | 76 | M | Infarction | 0 / 0 / 0 | 0 | 0 | No neglect | 0 | 2,224 |
| C9 | (-) | 72 | M | Hemorrhage | 0 / 0 / 0 | 0 | 0 | No neglect | 0 | 38,120 |
| C10 | (-) | 82 | M | Infarction | 0 / 0 / 0 | 0 | 0 | No neglect | 0 | 1,904 |
| C11 | (-) | 51 | F | Infarction | 0 / 0 / 0 | 0 | 0 | No neglect | 0 | 9,120 |
| C12 | (-) | 87 | F | Infarction | 0 / 0 / 0 | 0 | 0 | No neglect | 0 | 17,504 |
| C13 | (-) | 55 | M | Hemorrhage | 0 / 0 / 0 | 0 | 0 | No neglect | 0 | 28,392 |

**Fig. S1.** Overlaid lesions from patients with unilateral spatial neglect (n=9).

Warmer colors represent larger numbers of patients with overlapping lesions.


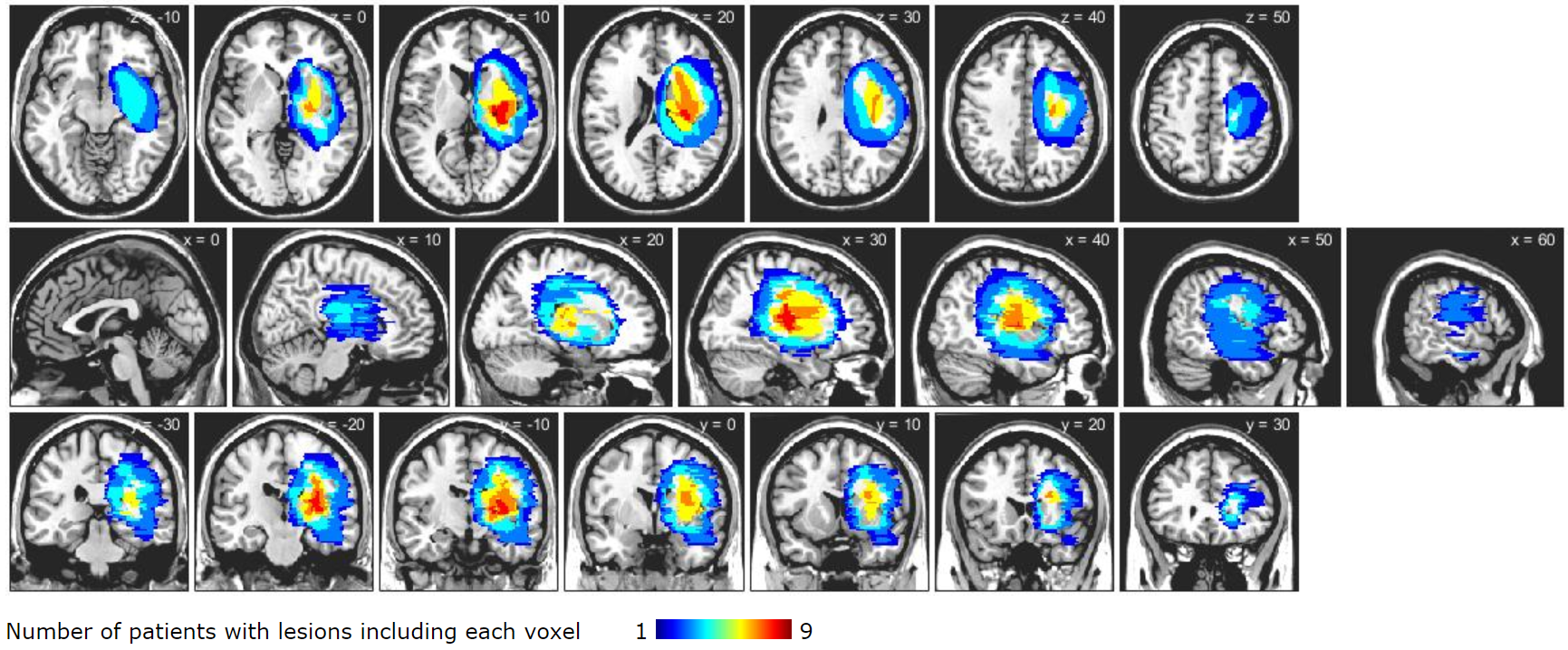


**Fig. S2.** Overlaid lesions from patients **without** unilateral spatial neglect (n=13).

Warmer colors represent larger numbers of patients with overlapping lesions.


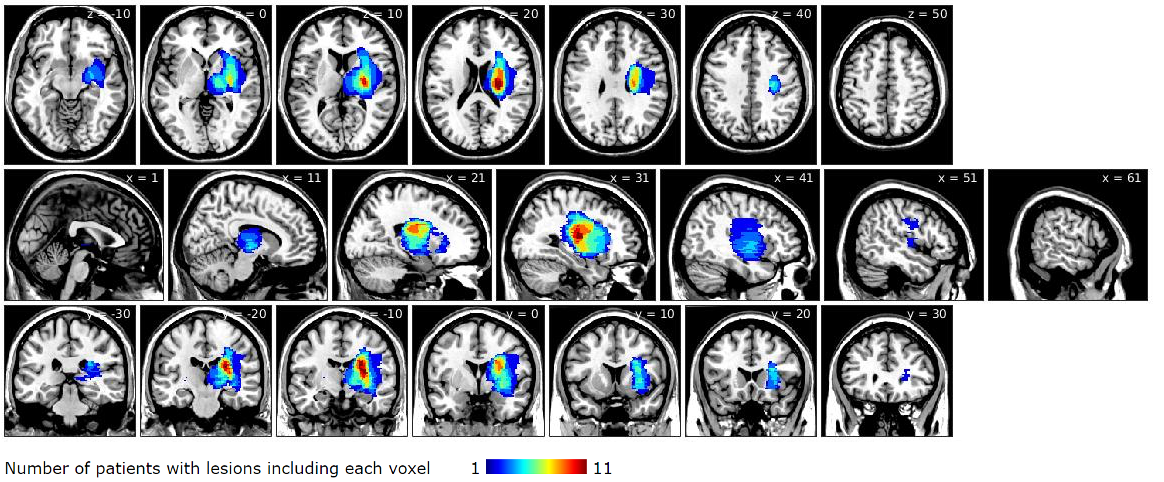


**Figure S3.** Histogram of white matter tract involvement.

The upper row shows the distribution observed in the USN(+) group, and the lower row shows the distribution observed in the USN(–) group. Each column represents one white matter tract.


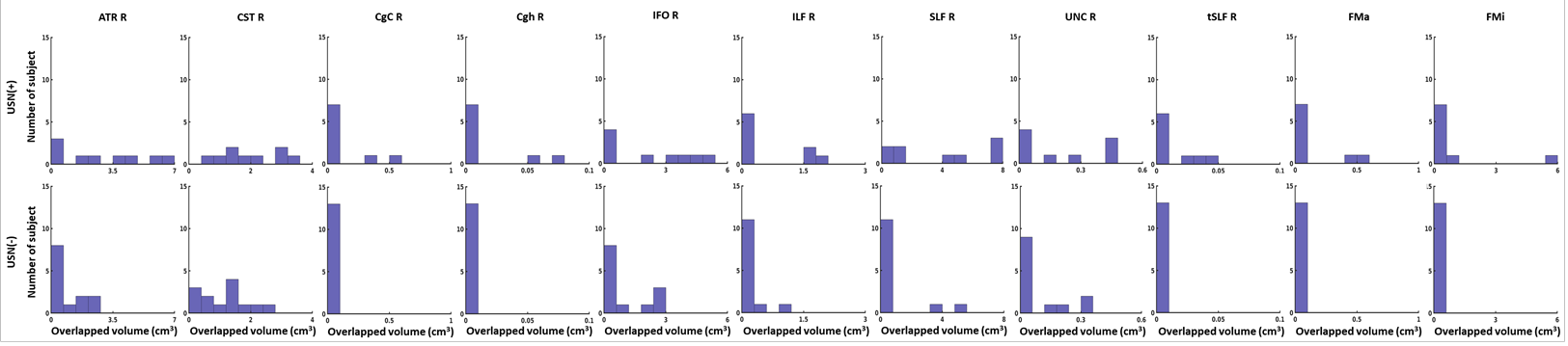


USN: unilateral spatial neglect, ATR: anterior thalamic radiation, CST: corticospinal tract, CgC: cingulum in the cingulate cortex, CgH: cingulum in the hippocampal area, IFO: inferior fronto-occipital fasciculus, ILF: inferior longitudinal fasciculus, SLF: superior longitudinal fasciculus, UNC: uncinate fasciculus, tSLF: temporal projection of the SLF, FMa: forceps major, FMi: forceps minor

**Table S2.** The correlation between the overlapped lesion volume of white matter tracts and the severity in unilateral spatial neglect.

|  | **White matter tractography atlas** | *r* | *p*-value |
| --- | --- | --- | --- |
| **The percentage of total uncrossed lines**  **in Albert test** | **Cingulum (cingulate gyrus) R** | -0.515 | 0.159 |
|  | **Forceps minor** | -0.374 | 0.317 |
|  | **Superior longitudinal fasciculus (temporal projection) R** | -0.365 | 0.335 |
| **The score of**  **Catherine Bergego scale** | **Cingulum (cingulate gyrus) R** | 0.822 | **0.012*** |
|  | **Forceps minor** | 0.897 | **0.002*** |
|  | **Superior longitudinal fasciculus (temporal projection) R** | 0.895 | **0.003*** |

*statistically significant results in the Spearman correlation analysis

**Figure S4.** Scatter plots between white matter tracts involvement and the severity of unilateral spatial neglect. (a) The percentage of total uncrossed lines in Albert test, (b) The score of Catherine Bergego scale.

| (a) | 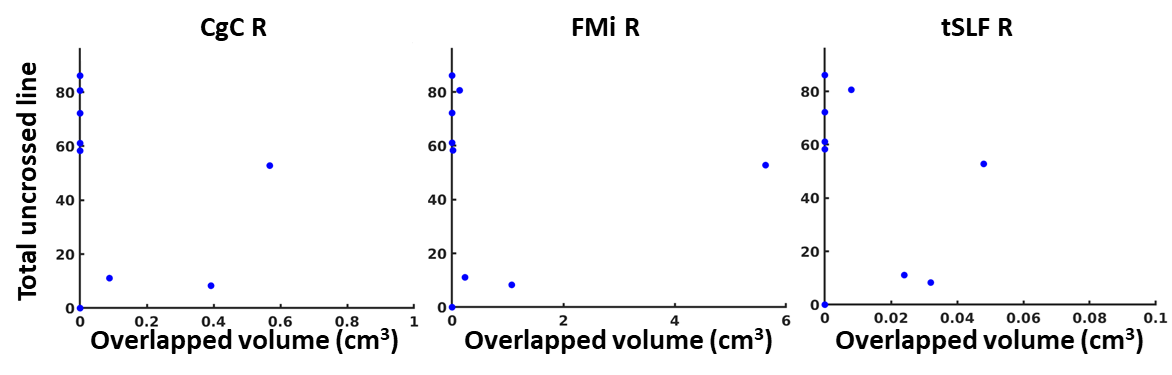 |
| --- | --- |
| (b) | 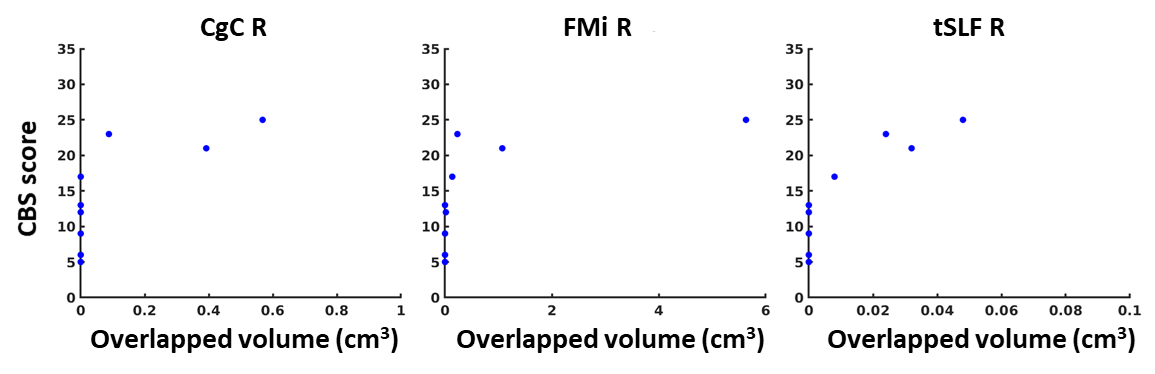 |
|  |  |

CgC: Cingulum in the cingulate gyrus, FMi: Forceps minor, tSLF: Temporal projection of the superior longitudinal fasciculus, CBS: Catherine Bergego scale
